# Prevalence of Insomnia in Type 2 Diabetes Mellitus Patients at a Tertiary Care Centre in Southern Kerala

**DOI:** 10.7759/cureus.109570

**Published:** 2026-05-24

**Authors:** Joe P John, Abraham Varghese, Sajit Varghese, Tomy Philip

**Affiliations:** 1 General Medicine, Pushpagiri Institute of Medical Sciences and Research Centre, Thiruvalla, IND

**Keywords:** comorbidities, insomnia, insomnia severity index (isi), sleep disturbances, type 2 diabetes mellitus

## Abstract

Objective: This study aimed to assess the prevalence, patterns, and associated factors of insomnia among patients with type 2 diabetes mellitus (T2DM) and to identify comorbid conditions that may contribute to sleep disturbances in this population.

Methodology: This cross-sectional study was conducted from 2022 to 2025 at a tertiary healthcare facility in Thiruvalla, Kerala, India. Using convenience sampling, 250 patients with T2DM were recruited. Patient demographic data, clinical history, and comorbidities were collected using a structured form. Insomnia was assessed using the Indian Insomnia Rating Scale (IIRS), a validated Malayalam-translated questionnaire. The IIRS scores were categorized as follows: 0-4, no insomnia; 5-10, mild insomnia; 11-20, moderate insomnia; and 21-30, severe insomnia. The T2DM was diagnosed according to the American Diabetes Association (ADA) criteria. Data analysis was performed using SPSS Statistics version 25 (IBM Corp., Armonk, NY, USA) to identify factors associated with insomnia in this population.

Results: Among the 250 participants, 141 (56.4%) were female, and 109 (43.6%) were male. Insomnia was reported in 101 (40.4%) participants, of whom 84 (83.2%) had secondary insomnia and 17 (16.8%) had primary insomnia. Difficulty falling asleep was reported by 48 (47.5%) participants, early awakening by 30 (29.7%), and difficulty maintaining sleep by 25 (24.8%). Duration of diabetes (p = 0.006), postprandial blood sugar (p = 0.002), and HbA1c (p = 0.002) were significantly associated with insomnia, whereas age, sex, occupation, and fasting blood glucose were not. The observed associations with glycemic parameters demonstrated small effect sizes.

Conclusion: Insomnia is common among patients with T2DM. The findings demonstrate an association between poor glycemic control and insomnia; however, causality cannot be established due to the cross-sectional study design. Healthcare providers should implement routine screening for insomnia, optimize glycemic control, address comorbid conditions such as obstructive sleep apnea and peripheral neuropathy, and provide sleep hygiene counseling as part of comprehensive diabetes management programs.

## Introduction

Insomnia is a commonly reported sleep disorder worldwide, characterized by difficulty falling asleep, maintaining sleep, or both. Approximately 10% of the population experiences insomnia severe enough to be classified as a medical diagnosis [1]. Insomnia may be secondary, arising in association with medical conditions, mental health disorders, or substance use, or primary, occurring without identifiable associated conditions [2]. Common symptoms include difficulty initiating sleep, frequent nighttime awakenings, and early morning awakenings, all of which can impair daytime functioning and reduce quality of life [3].

Type 2 diabetes mellitus (T2DM) is a complex metabolic disorder with significant global health implications [4]. Epidemiological evidence indicates a bidirectional relationship between sleep disturbances and metabolic dysregulation, including T2DM [5]. Poor sleep quality, short sleep duration, and chronic insomnia have been identified as modifiable risk factors for developing T2DM and may adversely affect glycemic control in patients with established diabetes [6]. Conversely, diabetes itself may contribute to the development of sleep disturbances through multiple mechanisms, including nocturia, peripheral neuropathic pain, glycemic fluctuations, autonomic dysfunction, inflammatory cytokine activation, and altered melatonin secretion, all of which may impair sleep quality and circadian rhythm regulation [5,6].

Despite growing recognition of the relationship between sleep disorders and T2DM, relatively few studies have comprehensively examined insomnia among diabetic patients across different regions of India [7]. There is a particular lack of regional data from Southern Kerala, India, regarding the prevalence, severity patterns, and clinical correlates of insomnia among individuals with T2DM [8]. This gap may partly reflect limited recognition of sleep disorders in routine diabetes care and the underutilization of structured sleep assessment tools in clinical practice [9].

In addition, several comorbid conditions frequently observed in patients with T2DM, including obstructive sleep apnea (OSA), hypertension, hypothyroidism, obesity, and peripheral neuropathy, may further complicate the evaluation of insomnia and contribute to sleep-related symptoms [6-8]. Understanding the coexistence of these conditions is important for improving clinical assessment and individualized management strategies in diabetic populations.

A clearer understanding of insomnia patterns in patients with T2DM may improve clinical screening, facilitate earlier identification of sleep disturbances, and support targeted interventions aimed at improving overall diabetes management and quality of life [10]. The primary objective of this study was to determine the prevalence of insomnia among patients with T2DM attending a tertiary care center in Southern Kerala. Secondary objectives included assessing insomnia severity patterns, associated sleep disturbances, comorbid conditions, and relationships between insomnia and glycemic parameters. The findings are intended to support the integration of routine sleep assessments into diabetes management practices in this region.

## Materials and methods

Study design and setting

This cross-sectional study was conducted to determine the prevalence of insomnia among patients with T2DM. The study was conducted at Pushpagiri Institute of Medical Sciences and Research Center in Thiruvalla (Kerala, IND), a tertiary healthcare facility with both general medicine and endocrinology departments. The study was conducted over 21 months following approval from the Institutional Ethics Committee (approval no. PIMSRC/E1/388A/43/2023). Patients were enrolled consecutively. All individuals who met the inclusion and exclusion criteria (Table 1) and presented to the study site during the study period were invited to participate. Written informed consent was obtained from all participants prior to enrollment.

**Table 1:** Inclusion and exclusion criteria T2DM: Type 2 diabetes mellitus

Category	Criteria
Inclusion criteria	Adults aged 18-80 years
	Diagnosed with T2DM per the American Diabetes Association (ADA) criteria [4]
	Provided written informed consent
Exclusion criteria	Patients with acute diabetic complications (diabetic ketoacidosis, hyperosmolar hyperglycemic state, hypoglycemia)
	Use of medications or substances affecting circadian rhythm (e.g., alcohol, narcotics)
	Presence of chronic debilitating conditions (e.g., cancer-related pain, post-stroke complications)
	Pregnant women
	Critically ill patients
	Patients with known psychiatric disorders

Sample size calculation

The study population included patients attending the endocrinology or general medicine outpatient departments, as well as those admitted to the general medicine inpatient unit. Using a previously reported insomnia prevalence of 30.6% from Otaka et al. [11], the minimum sample size was calculated using the formula:

\[ n = \frac{Z_{\alpha}^2 \, P \, Q}{d^2} \]

where $Z_{\alpha} = 1.96$, $P = 0.306$, $Q = 0.694$, and $d = 20\% \text{ of } P = 0.0612$. Thus, the calculated minimum sample size was $n = 218$, and a total of 250 T2DM patients were ultimately included in the study.

Data collection

Data were collected using a structured pro forma administered by trained interviewers to capture participants' demographic and clinical characteristics. Information regarding clinical history, physical examination findings, comorbidities, and laboratory parameters, including fasting plasma glucose (FPG), postprandial blood sugar (PPBS), and glycated hemoglobin (HbA1c), was recorded systematically. 

Insomnia was assessed using the Indian Insomnia Rating Scale (IIRS), a validated Malayalam-translated questionnaire developed for the Indian population [12]. The IIRS was used consistently throughout the study for both screening and severity assessment of insomnia. Based on the obtained scores, insomnia severity was classified as no insomnia (0-4), mild insomnia (5-10), moderate insomnia (11-20), and severe insomnia (21-30). Patients with an IIRS score of ≥5 were categorized as having insomnia. The diagnosis of T2DM was confirmed according to the American Diabetes Association (ADA) criteria [4], using FPG, two-hour PPBS, OGTT, HbA1c, or random plasma glucose values.

Definition of primary and secondary insomnia

Primary insomnia was defined as insomnia occurring without a clinically documented medical condition known to contribute significantly to sleep disturbance. Secondary insomnia was defined as insomnia occurring in the presence of clinically documented comorbid medical conditions potentially associated with sleep disturbance, including OSA, peripheral neuropathy, hypothyroidism, chronic pain conditions, or other chronic systemic illnesses. Classification was based on the coexistence of these conditions rather than establishing direct causality or temporal sequence.

Assessment of OSA

The presence of OSA was identified from previously documented clinical diagnoses available in the patients' medical records. Polysomnography was not performed as part of the study protocol. The treatment adequacy of OSA was not systematically assessed during the study period.

Data sources and variables

Eligible patients were interviewed using structured pro formas. Demographic variables included age, sex, and occupation. Clinical variables included duration of diabetes, glycemic parameters (FPG, PPBS, HbA1c), and associated comorbidities such as hypertension, hypothyroidism, peripheral neuropathy, cardiac disease, respiratory disease, chronic kidney disease, chronic liver disease, and OSA. Mild sleep-related symptoms not fulfilling the diagnostic threshold for insomnia according to IIRS scoring criteria [12] were categorized as subclinical sleep disturbances.

Statistical analysis

Data were coded and entered into Microsoft Excel (Microsoft Corp., Redmond, WA, USA), then analyzed using SPSS Statistics version 25 (IBM Corp., Armonk, NY, USA). Descriptive statistics, including frequencies, percentages, and means ± standard deviation (SD), were calculated. The prevalence of insomnia was reported as a percentage with 95% confidence intervals. Associations between insomnia and categorical variables were assessed using the chi-square test. Associations with continuous variables were evaluated using independent t-tests or nonparametric tests, as appropriate. Statistical significance was defined as a p-value < 0.05.

## Results

Among the 250 participants, 141 (56.4%) were female, and 109 (43.6%) were male. Insomnia was identified in 101 (40.4%) participants based on an IIRS score ≥5. Of these, 84 (83.2%) were classified as having secondary insomnia and 17 (16.8%) as having primary insomnia (Table 2).

**Table 2:** Demographic and clinical profile of participants OSA: Obstructive sleep apnea

Variable	Category	Frequency	Percentage (%)
Age	< 35	8	3.2
	35-45	26	10.4
	45-55	57	22.8
	55-65	99	39.6
	≥ 65	60	24.0
Sex	Male	109	43.6
	Female	141	56.4
Occupation	Professional	3	1.2
	Semi-professional	46	18.4
	Clerical/shop/farm	38	15.2
	Skilled	55	22
	Unskilled	108	43.2
Hypertension	Present	123	49.2
Hypothyroidism	Present	49	19.6
OSA	Present	34	34.0
Peripheral neuropathy	Present	50	20.0
Cardiac conditions	Present	23	9.2
Respiratory conditions	Present	18	7.2
Chronic kidney disease	Present	3	1.2
Chronic liver disease	Present	3	1.2

In primary insomnia, 10 (58.82%) patients had mild insomnia, followed by five (29.41%) with moderate and two (11.77%) with severe insomnia. In secondary insomnia, 40 (47.62%) patients each had mild and moderate insomnia, while severe insomnia was observed in four (4.76%) patients (Table 3).

**Table 3:** Comorbidities in primary vs. secondary insomnia Secondary insomnia classification was based on the coexistence of clinically documented comorbid conditions potentially associated with sleep disturbance, including OSA, peripheral neuropathy, hypothyroidism, and other chronic medical illnesses. OSA: Obstructive sleep apnea

Severity of insomnia	Type of insomnia	
	Primary	Secondary
Mild	10 (58.82%)	40 (47.62%)
Moderate	5 (29.41%)	40 (47.62%)
Severe	2 (11.77%)	4 (4.76%)
Total	17	84

Among patients diagnosed with insomnia (n = 101), disturbed sleep onset was reported in 48 (47.52%) patients, disturbed sleep maintenance in 25 (24.75%), and early awakening in 30 (29.70%). In contrast, these symptoms were substantially less frequent among participants without insomnia (Table 4).

**Table 4:** Sleep disturbances in insomnia vs. non-insomnia patients

Sleep disturbance	Insomnia present	Insomnia absent
Disturbed sleep onset	48 (47.52%)	6 (4.03%)
Disturbed sleep maintenance	25 (24.75%)	5 (3.36%)
Early awakening	30 (29.70%)	4 (2.68%)

In primary insomnia (n = 17), 9 (52.94%) patients had disturbed sleep maintenance, followed by seven (41.18%) with early awakening and five (29.41%) with disturbed sleep onset. In secondary insomnia (n = 84), 43 (51.19%) patients had disturbed sleep onset, followed by 23 (27.38%) with early awakening and 16 (19.05%) with disturbed sleep maintenance (Table 5).

**Table 5:** Sleep disturbances in primary vs. secondary insomnia

Disturbance	Primary, n (%)	Secondary, n (%)
Disturbed sleep onset	5 (29.41%)	43 (51.19%)
Disturbed sleep maintenance	9 (52.94%)	16 (19.05%)
Early awakening	7 (41.18%)	23 (27.38%)

There was no significant association between insomnia and sex (χ² = 1.71, df = 1, p = 0.191, Cramér’s V = 0.08) or occupation (χ² = 4.76, df = 3, p = 0.191, Cramér’s V = 0.13). As expected, core insomnia-related symptoms, including disturbed sleep onset, sleep maintenance difficulty, and early awakening, demonstrated statistically significant associations with insomnia status, reflecting their diagnostic relevance within the IIRS assessment framework. Therefore, these findings should be interpreted as supportive descriptive associations rather than independent predictive factors. Sleep onset disturbance (χ² = 18.92, df = 1, p < 0.001, Cramér’s V = 0.28), sleep maintenance disturbance (χ² = 22.45, df = 1, p < 0.001, Cramér’s V = 0.31), and early awakening (χ² = 16.37, df = 1, p < 0.001, Cramér’s V = 0.26) showed statistically significant associations with insomnia (Table 6).

**Table 6:** Association of patient characteristics with insomnia (chi-Square test) df: Degrees of freedom

Variable	Chi-sqaure test value	df	Effect size	P-value
Sex	1.71	1	0.08	0.191
Occupation	4.76	3	0.13	0.191
Sleep onset disturbance	18.92	1	0.28	<0.001
Sleep maintenance disturbance	22.45	1	0.31	<0.001
Early awakening	16.37	1	0.26	<0.001

There was no significant difference in age or FBS between patients with and without insomnia. However, statistically significant differences were observed for duration of diabetes, PPBS, and HbA1c levels, with higher mean ranks observed among patients with insomnia. Although these associations were statistically significant, the observed effect sizes were relatively small (r = 0.17-0.19), suggesting modest clinical effect strength (Table 7). The higher mean HbA1c values among participants with insomnia suggest poorer glycemic control in this subgroup, although the observed effect size remained small.

**Table 7:** Association of continuous variables with insomnia (Mann-Whitney U test) *Indicates statistically significant p-value. PPBS: Postprandial blood sugar,  FBS: Fasting blood sugar, HbA1C: Glycated hemoglobin

Variables	Insomnia present (mean ± SD)	Insomnia absent (mean ± SD)	Mean rank	Mann–Whitney U	p-value
Age	57.8 ± 10.2	55.9 ± 11.1	135.24 vs 118.90	6541.0	0.079
Duration of diabetes mellitus	11.4 ± 5.8	8.7 ± 4.9	140.77 vs 115.15	5982.5	0.006*
FBS	146.5 ± 42.1	139.2 ± 38.4	116.78 vs 105.44	5167.0	0.194
PPBS	228.6 ± 54.3	201.4 ± 49.8	125.96 vs 99.28	4359.5	0.002*
HbA1C	8.4 ± 1.2	7.8 ± 1.1	125.96 vs 99.28	4357.0	0.002*

## Discussion

The present cross-sectional study evaluated the prevalence, severity patterns, and associated clinical factors of insomnia among patients with T2DM attending a tertiary care center in Southern Kerala. The primary objective was to determine the prevalence of insomnia in this population, while secondary objectives included assessment of insomnia severity, associated sleep disturbances, comorbidities, and relationships with glycemic parameters. Among the 250 participants included in the study, 40.4% were found to have insomnia, highlighting the substantial burden of sleep disturbances among individuals with T2DM in this region.

The mean age of participants was 56.74 ± 10.74 years, which is consistent with the commonly affected age group for chronic metabolic disorders. Females constituted a slightly higher proportion of the study population (56.4%), similar to findings reported by Talwalkar et al. [13] and Batool et al. [14]. However, no statistically significant association was observed between sex and insomnia in the present study. Zhang et al. [15], in contrast, reported a higher prevalence of insomnia among middle-aged males, suggesting that demographic and sociocultural differences may influence sleep-related outcomes across populations.

The prevalence of insomnia observed in this study (40.4%) was comparable to findings reported in earlier studies. Talwalkar et al. [13] reported insomnia in 53.4% of participants, Otaka et al. [11] reported 30.6%, and Narisawa et al. [16] documented sleep disturbances in 43.9% of diabetic patients. Variability in prevalence estimates across studies may reflect differences in study populations, insomnia assessment tools, glycemic control profiles, healthcare access, and the coexistence of chronic comorbid conditions.

A notable observation in the present study was that the majority of insomnia cases (83.2%) were categorized as secondary insomnia. In this study, secondary insomnia was defined based on the coexistence of clinically documented medical conditions potentially associated with sleep disturbance rather than establishing a direct temporal or causal relationship. Common associated comorbidities included OSA, peripheral neuropathy, hypothyroidism, hypertension, and chronic systemic illnesses. These comorbid conditions may contribute to sleep disruption through pain, respiratory instability, metabolic dysregulation, nocturnal symptoms, and circadian rhythm disturbances.

The relationship between insomnia and T2DM is likely bidirectional. While poor glycemic control may contribute to sleep disturbances through nocturia, neuropathic pain, inflammatory cytokine activation, and autonomic dysfunction, chronic insomnia itself may adversely affect glucose metabolism by increasing cortisol secretion, sympathetic nervous system activation, systemic inflammation, and insulin resistance. Therefore, the associations observed in this study should not be interpreted as evidence of a unidirectional causal relationship.

No statistically significant association was observed between insomnia and demographic variables such as age, sex, or occupation. However, significant associations were identified between insomnia and duration of diabetes, PPBS, and HbA1c levels. Participants with insomnia demonstrated higher mean HbA1c values compared to those without insomnia, suggesting relatively poorer glycemic control among affected individuals.

Although these associations reached statistical significance, the observed effect sizes were relatively small (r = 0.17-0.19), indicating modest clinical effect strength. Therefore, the clinical significance of these findings should be interpreted cautiously. Nevertheless, even modest deterioration in sleep quality may contribute cumulatively to adverse metabolic outcomes over time in patients with chronic diabetes.

Otaka et al. [11] did not observe a significant association between HbA1c and insomnia, whereas Batool et al. [14] reported associations between sleep quality and educational status. These variations across studies emphasize the multifactorial nature of insomnia in patients with T2DM and suggest that sleep disturbances may be influenced by demographic, behavioral, psychosocial, and metabolic factors.

Primary insomnia in the present study was more commonly associated with sleep maintenance difficulty and early awakening, whereas secondary insomnia was more frequently associated with disturbed sleep onset. These findings may reflect the influence of comorbid medical conditions such as neuropathy, OSA, and hypothyroidism on nocturnal discomfort, respiratory stability, and circadian rhythm regulation.

Mild sleep-related symptoms not fulfilling the diagnostic threshold for insomnia according to IIRS scoring criteria [12] were categorized as subclinical sleep disturbances. These symptoms were present in a small subset of participants and may represent early or intermittent sleep dysfunction rather than clinically significant insomnia. Following reviewer feedback, the symptom distribution tables and data presentation were re-evaluated and corrected to avoid overestimation of symptom prevalence among non-insomnia participants.

Among the associated comorbidities observed in the study population, hypertension (49.2%), OSA (34%), and peripheral neuropathy (20%) were the most frequent. The OSA status was based on previously documented clinical diagnoses available in patient medical records; polysomnography was not performed as part of the study protocol. In addition, the adequacy of OSA treatment was not systematically evaluated, which may have influenced insomnia-related symptom reporting. The coexistence of OSA and insomnia may complicate clinical assessment because untreated OSA can mimic or exacerbate symptoms of insomnia.

The findings of this study support the growing recognition that sleep disorders are clinically relevant yet frequently underrecognized among individuals with T2DM. Routine screening for insomnia and associated sleep disturbances may help identify patients at risk of poorer glycemic control and reduced quality of life. Potential interventions may include optimization of glycemic control, management of comorbid conditions such as OSA and peripheral neuropathy, sleep hygiene counseling, behavioral sleep interventions, and referral for specialist sleep evaluation when clinically indicated.

Limitations

This study has several limitations that should be considered while interpreting the findings. First, the cross-sectional study design limits the ability to establish causal relationships between insomnia and T2DM; therefore, only associations can be inferred. Second, the use of convenience sampling from a single tertiary care center in Southern Kerala may limit the generalizability of findings to broader community populations. Because the study was conducted in a tertiary referral center, the sample may have included patients with more severe disease, multiple comorbidities, or poorer glycemic control, potentially leading to overestimation of insomnia prevalence compared to primary care settings. Third, the insomnia assessment was based on the self-reported IIRS, which may be subject to recall and reporting bias. Objective sleep assessment tools such as polysomnography were not used. Furthermore, OSA diagnoses were based on prior clinical documentation rather than standardized sleep studies conducted during the study period. The study also did not systematically assess treatment adequacy for OSA, depression, obesity, medication effects, physical activity, dietary habits, or psychosocial stressors, all of which may influence sleep quality and glycemic control.

Although multiple comorbidities were documented, multivariate regression analysis was not performed to adjust for potential confounding variables because of limited subgroup sizes for certain conditions. Additionally, the observed associations between glycemic parameters and insomnia demonstrated relatively small effect sizes, and the study did not evaluate dose-response relationships between insomnia severity and HbA1c levels. Finally, longitudinal follow-up was not performed; therefore, the temporal relationship between worsening glycemic control and progression of insomnia could not be determined.

## Conclusions

Insomnia was highly prevalent among patients with T2DM in this tertiary care population, with most cases occurring as secondary insomnia associated with comorbid medical conditions. Significant associations were observed between insomnia and longer duration of diabetes, elevated PPBS, and higher HbA1c levels, suggesting a relationship between poor glycemic control and sleep disturbances. However, the observed effect sizes were modest, and causal relationships cannot be established due to the cross-sectional study design. These findings emphasize the importance of routine screening for insomnia and associated sleep-related comorbidities in patients with T2DM to support comprehensive diabetes management and improve overall quality of life.
